# Improving hardiness among university students: A meta-analysis of intervention studies

**DOI:** 10.3389/fpsyg.2022.994453

**Published:** 2023-01-12

**Authors:** Gao Jianping, Zhu Zhihui, Samsilah Roslan, Zeinab Zaremohzzabieh, Nur Aimi Nasuha Burhanuddin, Soh Kim Geok

**Affiliations:** ^1^Faculty of Educational Studies, Universiti Putra Malaysia, Serdang, Malaysia; ^2^Institute for Social Science Studies, Universiti Putra Malaysia, Serdang, Malaysia

**Keywords:** hardiness, interventions, meta-analysis, university students, higher education

## Abstract

**Introduction:**

Increasing the hardiness of students is a crucial objective in higher education. Universities and colleges have created a variety of interventions to improve students' overall hardiness.

**Methods:**

In terms of the effects of such interventions, empirical research has shown inconclusive results. This meta-analysis applies 12 effect sizes from 12 independent empirical studies, with a total of 640 participants, to assess the overall impact of interventions on students' hardiness and to test for moderators, in light of the contradictory findings in prior work. The current meta-analysis calculates the standardized mean differences (SMD) of pre-post interventions. The level of study heterogeneity, represented by *I*^2^, was interpreted as small (*I*^2^ ≤ 25%), moderate (25% < *I*^2^ ≤ 50%), substantial (50% < *I*^2^ ≤ 75%), or considerable (*I*^2^ > 75%). Twelve studies met the inclusion criteria and were included in the meta-analysis.

**Results:**

The results show that the interventions had a significant positive overall effect on students' hardiness (*g* = 0.998, *k* = 12) and show significant heterogeneity among effect sizes. Among the interventions, cognitive-based intervention yielded the largest mean effect size (*g* = 2.015, *k* = 5). Furthermore, moderator analyses suggest that the effects of the interventions on students' hardiness are moderated by respondent type, culture, intervention type, research design, years, and duration of intervention.

**Discussion:**

We conclude that interventions that promote students' hardiness are officious. Despite the low homogeneity of the results and limitations of this meta-analysis (e.g., a small number of included studies) which might have influenced the findings, the large fail-safe N suggests that these findings are robust. The study examined potential causes of heterogeneity and emphasized the importance of further research in this area.

## 1. Introduction

The transition from high school to college is riddled with complications (Ribeiro et al., 2018). Students go through significant changes during this transition, such as the formation of new social networks and responsibilities; social displacements, such as those experienced by members of racial minorities in university populations; a lack of structure in university activities; a lack of preparation; and new academic responsibilities (Johnson et al., 2011; Briggs et al., 2012; Thomas, 2012; Taylor et al., 2014; Wrench et al., 2014). These challenges are exacerbated, since higher education (HE) places a high value on student responsibility, thus resulting in a plethora of conflicting expectations that include balancing academics, family, and leisure (Wrench et al., 2014). Academic demands may result in students having a gap between their expectations and the reality of HE, resulting in difficulty to adapt and a high sensitivity to stress (Stephens and Gunther, 2016; Ribeiro et al., 2018).

Studies in the literature on coping include mostly explore the various facets of how university students adapt to or deal with change, challenges, and traumatic experiences. One of many possible avenues of exploration in this area involves personality structures that are hypothesized to serve as buffers against the detrimental psychological and physical effects of stress. The benefits of identifying such constructs are clear, as training, encouragement, or otherwise focusing on them may lead to an improved quality of life. Hardiness has emerged as a crucial factor in mitigating or resisting the impacts of stress (Yang et al., 2022). Three essential components make up a hardy personality: control, commitment, and challenge (Wardani, 2020). These three elements work together to help students succeed in difficult situations. According to a study on the development of hardiness by Kobasa et al. (1982), people become hardy if they have a range of experiences as youngsters. Individuals can receive intellectual and social knowledge through these experiences. Hardiness research has discovered that individuals with hardiness features do not give up readily under duress, fall ill less frequently, and may behave adaptively when stressed (Stein and Bartone, 2020).

While there has been extensive and sometimes promising hardiness research in the past 20 years, researchers explored the effectiveness of several interventions on hardiness in various clinical and non-clinical settings (e.g., Macedo et al., 2014). University students differ from other populations in that their appraisals and adaptations to their university environment shape their hardiness (Cheng et al., 2019). Evidence on interventions for hardiness enhancement that has been obtained from other populations is not directly applicable to these students. Compared to the general population, students showed lower levels of hardiness (Kowalski and Schermer, 2019), and their hardiness was found to be negatively correlated with their psychological distress (Abdollahi et al., 2018b). Few interventions are specifically designed to enhance the hardiness of university students (Maddi, 2002; Maddi and Harvey, 2006). Furthermore, the findings for the effectiveness of such interventions are inconsistent (Maddi, 2002; Maddi and Harvey, 2006). Given the potential role of hardiness in the prevention of stress-related disorders and the alleviation of psychological distress (Abdollahi et al., 2015), exploring the interventions for hardiness enhancement among university students will be beneficial for promoting their physical and psychological wellbeing.

To the best of our knowledge, there is currently a considerable gap in the research, because there is no current synthesis of interventions aimed at enhancing hardiness. By offering a meta-analysis of the studies on interventions' effects on university students' hardiness, the present researchers seek to advance the existing research with their work. The third wave of hardiness research focuses on the creation and assessment of interventions that seek to create or strengthen psychological interventions and avoid stress-related mental dysfunctions (Waite and Richardson, 2004; Bengel and Lyssenko, 2012). As such, the following research questions (RQ) were investigated:

RQ 1: What is the general impact of diverse interventions on the hardiness of university students (overall effect)?
RQ 2: Is the variance in the effects among research [heterogeneity in effect sizes (ESs)] statistically significant?

## 2. Literature review

### 2.1. Hardiness: Its structure

Hardiness is linked to effective coping mechanisms, including the reduction of risk perception and raising one's likelihood to conquer challenges (Cole et al., 2004). These personal qualities influence more positive and proactive behaviors (Johnsen et al., 2017). According to Kobasa (1979), the ability to withstand, provide resistance against, and recover from stressful events is correlated to hardiness. Hardiness is a personality trait made up of the elements of control, challenge, and commitment (Maddi et al., 2012). Thus, “control” refers to people's convictions that they can regulate their emotions in the face of academic difficulties to attain their professional goals (Abdollahi et al., 2018a). People who are strongly in control prefer to exert some influence over the results, rather than being helpless and unresponsive (Maddi, 1999; Maddi et al., 2012). Hardy control encourages a person to regard their stresses as being modifiable, which increases their incentive to participate in effortful coping (Maddi, 2002). Commitment is an individual's devotion to activities that are significant and engaging to that individual, such as employment, sport, academics, religion, or pastimes (Huang, 2015). It is the motivation that allows a person to remain connected with people and events, rather than living in loneliness and isolation (Maddi et al., 2012). The interpretation of a person's work demands as chances for their personal development is referred to as a “challenge” (Bakker and de Vries, 2021). People who can overcome obstacles view pressures as normal and a chance to advance, rather than an opportunity to run away from difficult situations (Travis et al., 2020).

### 2.2. Measurements of hardiness

In the early years of working with the notion, measuring hardiness was extremely difficult. Kobasa (1979) first analyzed it using an aggregation of 18 distinct psychological measures, aiming at capturing the aspects of commitment, control, and challenge. To make the practical use of hardiness simpler, Maddi (1987) developed a method to teach hardiness skills. In order to deal with stressful situations efficiently, the first hardiness education program incorporated “cognition, emotion, and action,” and the feedback from this process was utilized to increase participants' commitment, challenge, and control (Maddi et al., 1998, p. 79). Building on earlier research on hardiness education (Maddi, 1987; Maddi et al., 1998) with working people, Maddi et al. (2002) assessed how well hardiness education improved high-risk undergraduate students' retention rates and GPA.

Furthermore, Bartone (1989) created a 50-item, more concise and coherent hardiness test using samples of bus drivers and telephone company administrators. The DRS was later condensed and enhanced in numerous ways, yielding 30-item and 15-item versions (Bartone, 2007). The DRS-15 has been widely tested in both military and non-military samples, with generally positive findings (Britt et al., 2001; Bartone et al., 2008; Andrew et al., 2013). The brief DRS-15's final version intended to increase scale reliabilities and minimize linguistic bias in item phrasing (Bartone, 2013). The revised DRS-15 shows solid psychometric properties (Hystad et al., 2010) and evidence of predictive validity (Johnsen et al., 2013; Bartone et al., 2016). The most recent version of the Personal Views Survey (the PVS-IHR) and the one presently being utilized in research is an 18-item scale. The PVS-IIIR is a compilation of the most dependable components from previous research. There is also a Health Related Hardiness Scale (HRHS) (Pollock, 1986) and a Family Hardiness Scale (FHS) (McCubbin et al., 1986), but research using these tools is minimal.

In this research, we concentrated on intervention studies that made use of one or both of the two most well-known and popular standardized tools for assessing hardiness: Lang et al. (2003) Hardiness Scale (LGHS) and the Personal Views Survey III-R (PVS III-R; Maddi and Khoshaba, 2001) and Academic Hardiness Scale (AHS). Benishek and Lopez (2001) established the term AHS, which has been used to explore why certain students are eager to embrace academic challenges, while others shun hard academic material for fear of damaging their academic performance. The academic hardiness scale was developed by researchers following Kobasa et al. (1982) concept. These tests are established evaluation instruments used to gauge students' hardiness. Since they were first made available to the public, these tests have undergone several validation examinations conducted according to strict measurement development criteria.

### 2.3. Interventions for hardiness

Many researchers have identified the choice of coping mechanism as a crucial behavioral factor that influences the health and functional outcomes of a hardy disposition. The coping mechanism may function, in part, as a behavioral indicator of hardiness. The transactional model of stress (e.g., Lazarus, 2000) outlined an iterative process, in which perceptions of danger, assessments of coping resources, and, finally, decision-making over coping responses interact to determine the subjective stress felt by an individual. An inference from this transactional and iterative process is that students may be able to become more resilient and interact with their environments with lower levels of subjective stress by learning to use different coping mechanisms, developing new perspectives on stressors, and finding meaning in circumstances they might otherwise avoid. It is still necessary to investigate, in greater depth, the processes through which hardiness may boost responsiveness to psychological intervention so that hardiness research can continue to enrich evidence-based applied practice (Martens, 1987).

### 2.4. Current empirical studies of hardiness in universities

Empirical research has been done to look at how interventions affect how resilient and hardy university students are. The instruments used by researchers, and the study methodologies used in these studies, share many commonalities. For instance, the majority of research used one or more standardized assessments to assess the potential improvement in students' hardiness. A pre-experimental approach, such as a one-group pre-test and post-test design, was employed by the majority of studies to examine changes in hardiness before and after the interventions (Weissinger, 2003; Burbach et al., 2004; McKown, 2004). A quasi-experimental design, with convenience samples, was also popularly employed (Dale and Ballotti, 1997). In terms of intervention and execution among researchers, there were more variances than similarities. For example, treatment length varied from a few weeks (McGregor, 2001) to many years (Scott et al., 1998; Magnussen et al., 2000; Spelic et al., 2001; Bartlett and Cox, 2002). According to Thompson and Rebeschi (1999), the students studied might be either undergraduates, graduates, or postgraduates.

It is not surprising to learn that the study outcomes are uneven, given the diversity of interventions, student groups, and implementations. Similar approaches have had varying outcomes in other studies. Jafar et al. (2016) discovered that certain interventions were successful, while others failed (e.g., Jameson, 2014). It is not possible to assess the effectiveness of the various strategies for strengthening the resilience and hardiness of university students just by looking at individual empirical research. It is extremely challenging to identify the potential causes of the conflicting results obtained from various research. The link between intervention and hardiness in HE settings needs to be investigated in light of the conflicting findings about the impact of interventions on university students and hardiness.

## 3. Methodology

### 3.1. Search strategy

In this section, the methodology utilized to extract papers related to different interventions to promote and improve hardiness, academic hardiness, and psychological hardiness in university students is discussed. The reviewers conducted a systematic review and meta-analysis; came up with the eligibility and exclusion criteria and review process stages (identification, screening, and eligibility); and conducted data abstraction and analysis using the PRISMA guideline. Searches were conducted on electronic databases, including PubMed, PsycINFO, Scopus, CINAHL, Embase, and ERIC. All studies, including reviews published previously, references listed, and related essays, were checked, retrieved, and assessed for possible inclusion in the review.

### 3.2. Study selection

There are pre-established eligibility and exclusion criteria. First, only studies that used pre-and post-intervention designs, randomized experimental designs, non-randomized experimental designs, or quasi-experimental designs were eligible for inclusion. Excluded studies included case reports, cross-sectional studies, surveys, case-control designs, and prospective cohort studies. Second, only papers written in the English language were included. Thirdly, as the review process focused on university students' hardiness, academic hardiness, and psychological hardiness, the authors of this study did not include studies on children, studies with school student or athlete populations, or studies that specified recruiting students at universities or colleges. Lastly, the primary outcome measured in this line of study was hardiness. Thus, hardiness should have been evaluated either pre- or post-intervention, or at least once after the intervention had ended for both intervention and comparison groups.

### 3.3. Coding of study variables

The author(s), year of publication, country, participants, study design, sample size, nature of the intervention, duration, and outcome variables were all used to extract and code information from the chosen studies. To decrease the likelihood of mistakes and identify moderating variables when possible, information from the chosen studies was retrieved and coded. The studies included are listed in Table 1.

**Table 1:** Intervention characteristics of the included studies.

**No**.	**References**	**Country**	**Participants**	**Design**	**N**	**Nature of intervention**	**Duration (weeks)**	**Outcome variables**
1	Antika et al., 2020	Indonesia	The guidance and counseling students	One-group PR–PO	46	Mind-skills training	NM	- Academic hardiness^*^
2	Fard and Moradkhani, 2018	Iran	University students	A semi-experimental study with a PR–PO control group	16 int/16 cont	ACT	4	- Hardiness^*^ - Procrastination^*^ - Frustration tolerance^*^
3	Nikoozadeh, 2020	Iran	University students with a high-stress score	One-group PR–PO	31	Hardiness Training Intervention	12	- Perceived stress^*^ -Psychological hardiness^*^
4	Torfayeh et al., 2020	Iran	University students	Quasi-experimental with PR-PO	17 int/17 cont	MCT	4	-Psychological hardiness^**^
5	Almahaireh et al., 2018	Jordan	University students	Quasi-experimental with PR-PO	15 int/15 cont	Preventive counseling program	8	- Psychological hardiness^*^ - The positive use of SNSs^*^
6	Kanekar et al., 2010	USA	Asian Indian International Students	A one-between/one-within subjects randomized comparison	39	An internet-based intervention	8	- Social support - Hardiness - Acculturation
7	White et al., 2020	USA	Military medical students	One-group PR–PO	68	Hyper-realistic surgical simulation training course	1	- Hardiness - Emotional intelligence
8	Toosang et al., 2021	Iran	Medical students	Quasi-experimental with PR/PO	100 int/100 cont	CBT	16	- Resilience^*^ - Psychological hardiness^*^
9	Khoiriyah et al., 2020	Indonesia	Final year students completing final project in IAIN Kudus	One group PR-PO	21	CRT^a^	6	- Hardiness^*^
10	Jafar et al., 2016	Iran	University students	Quasi-experimental with PR/PO	15 int/15 cont	Group training of CBT-based stress management	10	- Anxiety^*^ - Psychological hardiness^*^ - General self-efficacy^*^
11	Sahranavard et al., 2019	Iran	Female medical students	Quasi-experimental with PR/PO	15 int/15 cont	Group training of CBT -based stress management	10	- Anxiety^*^ - Hardiness^*^ - Self-efficacy^*^
12	Jameson, 2014	USA	Junior baccalaureate nursing students	Quasi-experimental PR/PO	40 int/39 cont	A hardiness educational intervention	6	- Hardiness - Perceived stress^*^

^*^Indicate significant effects on outcomes; int, Intervention; cont, Control; ACT, Acceptance and Commitment Therapy; CBT, Cognitive Behavioral Therapy; CRT, Cognitive Restructuring Technique; MCT, Metacognitive Therapy; PR, Pre-test; PO, Post-test; SNS, social networking sites; NM, not mentioned.

^a^CRT is a core part of CBT. ^**^p ≤ 0.01.

### 3.4. Systematic review process

**Figure 1:**
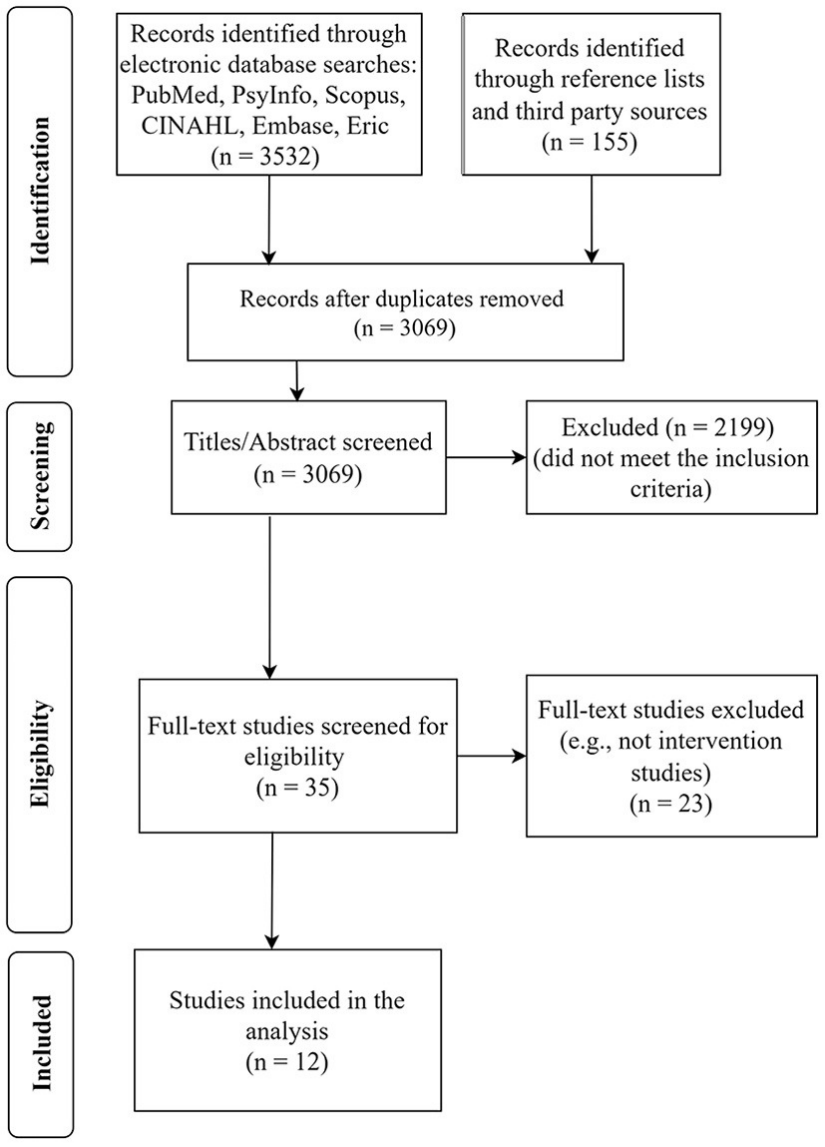
The flow diagram of the study (adopted from Moher et al., 2009).

The review process was performed in March 2022. The first phase identified the keywords used for the search process. The current study used search terms related to students (e.g., “college student,” OR “university student,” OR “undergraduate”), hardiness (e.g., “academic hardiness,” OR “psychological hardiness,” OR “hardiness,”), and interventions (“treatment,” OR “training,” OR “therapy,” OR “intervention”). At this stage, after careful screening, unconnected research, studies with insufficient data, duplicate sources, and studies with unclear methodology were removed. Thirty five studies were left for full-text analysis after the second screening of titles and abstracts. Twenty three studies were not included at this stage because they were not intervention studies or their population didn't focus on university students. Finally, 12 controlled studies on hardiness interventions for university students were deemed to be appropriate for the meta-analysis (Figure 1).

### 3.5. Study reporting quality

The chosen studies' respective qualities were assessed using the current criteria developed for examining quantitative study data (Kmet et al., 2004). The quality of research was assessed using multiple criteria, including determining the appropriate sample size, giving adequate information for outcomes, and assessing variation in the major results. The ratings of the two independent reviewers varied from 0.89 to 1 (the first researcher obtained a mean of 0.97, while the second obtained 0.96; a range of 0–1). All studies had the same overall score from both reviewers.

### 3.6. Statistical analyses

To conduct the meta-analysis for hardiness, academic hardiness, and psychological hardiness, this study employed the Comprehensive Meta-Analysis (CMA) software version 2.2 (Biostat, NJ, USA). To calculate ESs, means and standard deviation (SD) calculations were employed. When means and SDs were unavailable, ESs were calculated using alternative statistics (e.g., t and F). There was one ES for each outcome variable when more than one instrument was utilized to measure the same outcome variable (Thalheimer and Cook, 2002). Hedges' g, together with the corresponding *p*-value and its 95 percent confidence interval (CI), served as the ES computed in each study. Because Hedges' g offers a less biased estimate of ES for small samples, it was selected (Hedges and Olkin, 1985). All outcomes' variables data were pooled for meta-analysis using the random-effects model. Cohen's (1988) paradigm of small (0.2), medium (0.5), and large (0.8) was used to evaluate ES magnitudes.

### 3.7. Heterogeneity

Because of the variations in samples, measures, and designs between studies, random-effects models were employed in all analyses to determine if observed heterogeneity between studies was systematic beyond what might be expected owing to sampling error. The Cochrane Q and *I*^2^ statistics were used to calculate the heterogeneity of the ESs. The Q-test assesses heterogeneity owing to sampling error, but it is insufficiently strong to detect actual heterogeneity. As a result, the *I*^2^ statistic was used to see if the proportion of variance between trials was attributable to heterogeneity, rather than chance owing to sampling error (Higgins and Thompson, 2002). The *I*^2^ value ranges from zero to one hundred percent. Indicated as no, low, medium, and high heterogeneity, respectively, were 0, 25, 50, and 75%. By deleting each study one at a time, a sensitivity analysis was also carried out to determine the influence of each study on the heterogeneity result (Borenstein et al., 2021).

### 3.8. Moderator analysis

Certain variables that differed among studies were investigated as moderators to measure heterogeneity across studies. Categorical factors, such as type of respondents, types of interventions, culture, and research designs, were subjected to subgroup analysis. For continuous variables, like year and duration, meta-regression was used. For all moderator analyses, mixed-effects models were used. A random-effects model was used to aggregate studies within each subgroup, while a fixed-effects model was used to look at differences between subgroups.

### 3.9. Publication bias

The funnel plot, Egger's test, and the fail-safe N were all used to examine publication bias. The funnel plot shows that the ESs will be distributed symmetrically around the mean if a meta-analysis incorporates all pertinent interventions and there is no publication bias (Light and Pillemer, 1984). Otherwise, the funnel plot will reveal an uneven distribution of ESs, indicating the presence of publication bias (Light and Pillemer, 1984). The asymmetry of the funnel plot is determined by Egger's test. The fail-safe N is the number of missing studies, with zero ESs necessary to convert a significant ES to a non-significant ES (Lipsey and Wilson, 2001).

## 4. Results

### 4.1. Study selection

In total, we found 3, 532 records: PubMed (923), PsycInfo (455), Scopus (806), CINAHL (260), Embase (603), ERIC (330), searching reference lists (151), and suggested by third parties (4). After duplicates were removed, 3, 069 records remained for screening. The present authors removed 2, 199 publications that did not fulfill the inclusion criteria, based on a screening of abstracts and titles. We then assessed 35 full-text articles. Twenty three studies were not included at this stage because they were not intervention studies, or because their populations did not focus on university students. Finally, 12 controlled studies on hardiness interventions for university students were deemed appropriate for meta-analysis (Figure 1).

### 4.2. Study characteristics

Table 1 provides an overview of the study's features. All of the studies that were considered were published between April 2014, and October 2021. The majority of the studies (9 in total) came from outside of the Western hemisphere. It should be emphasized that quasi-experimental research, using a pre-test-post-test design, was employed in six different investigations. The studies included 640 participants. Sample sizes ranged from 21 to 200. The durations of the interventions varied between 1 week and 16 weeks. One study did not report its duration. Notably, two researches (Jafar et al., 2016; Sahranavard et al., 2019) evaluated their outcome variables using group training in CBT-based stress management. Six studies assessed hardiness (Kanekar et al., 2010; Jameson, 2014; Fard and Moradkhani, 2018; Sahranavard et al., 2019; Khoiriyah et al., 2020; White et al., 2020), five studies evaluated psychological hardiness (Jafar et al., 2016; Almahaireh et al., 2018; Nikoozadeh, 2020; Torfayeh et al., 2020; Toosang et al., 2021), and one research assessed academic hardiness (Antika et al., 2020).

### 4.3. Effect size and homogeneity testing

**Figure 2:**
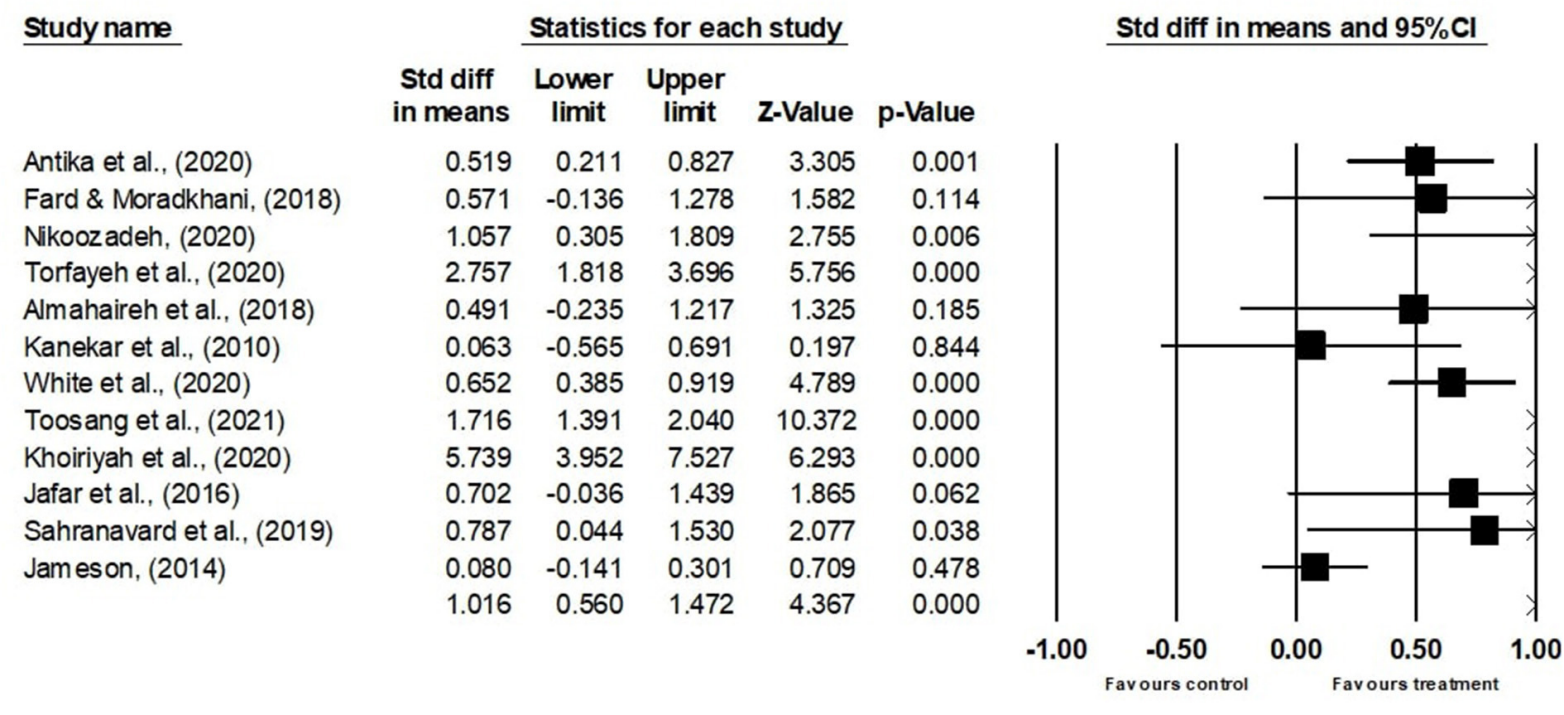
Forest plot for the random-effects model.

Because of the variance between studies in terms of samples, measures, and designs, random-effects models were used for all analyses; and to assess if the observed heterogeneity between studies was systematic beyond what could be expected due to sampling error (Viechtbauer, 2007). Table 2 shows the results of the overall ES analysis and the test for heterogeneity. When all 12 ESs are combined, the overall estimate of ES is 0.998, with a standard error of 0.345. This indicates that, on a large ES, the intervention increased students' scores on standardized hardiness tests by 0.998 SDs. As this ES is high, it is statistically significant (*z*-value = 4.367, *p* < 0.001). The 95% CI of the mean ES is (0.549, 1.446), indicating that, 95% of the time, the true mean ES of instructional intervention will fall between 0.549 and 1.446. The Forest plot illustrates a series of estimates and their CIs at a percentage of 95% (Figure 2). Each study's ES (outcome) is also shown by a square per box, and their CIs are represented through horizontal lines. This plot displays broader CIs and inconsistent response rates, clearly demonstrating the heterogeneity of the chosen studies.

**Table 2:** Overall mean ES summary and test for heterogeneity.

				**Homogeneity test**			**Tau-squared**			**Test of null (2-tailed)**	
**k**	***N***	**Hedges' g**	**95% CI**	**Q(g)**	***p***	***I* ^2^**	**Tau^2^**	**SE**	**Tau**	**Z**	***p***
12	640	0.998	[.549,1.446]	123.147	0.000	91.068	0.526	0.345	0.725	4.367	0.000

K, number of effect sizes; CI, confidence interval.

A Q statistic of 123.147 and an anticipated value of 11 were obtained from the test for heterogeneity. A *p*-value of < 0.001 indicates that the test of the null hypothesis is statistically significant. This suggests that an explanation is required for the statistically significant variability of the ESs. The estimated *I*^2^ value is 91.068%, indicating that the actual variations in the ES account for 91.068% of the observed total variance between studies. The variance of the mean ES is 0.526 (*T*^2^ statistic) and the SD is 0.725 (*T*-statistic).

### 4.4. Publication bias

**Figure 3:**
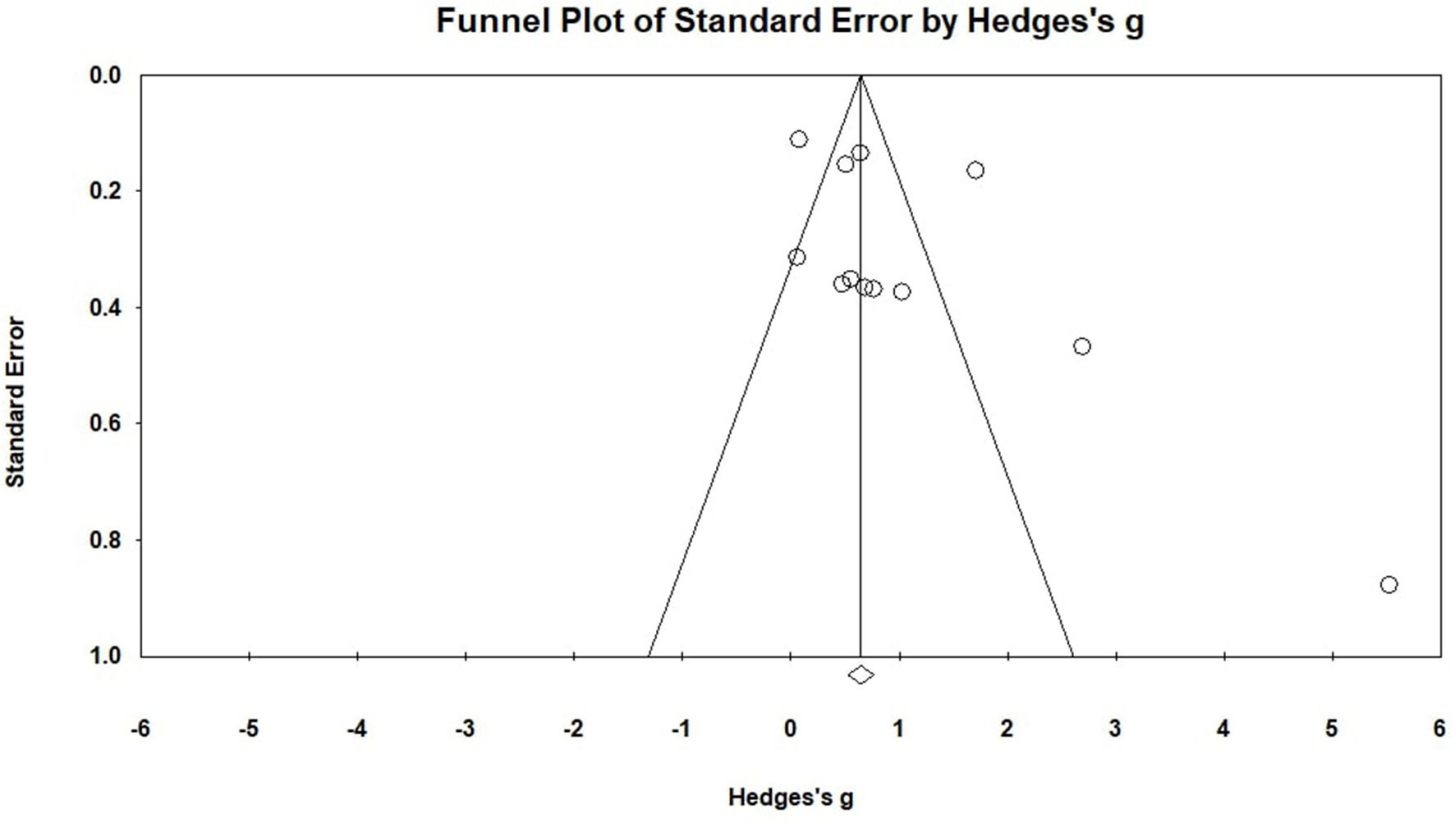
Funnel plot of the overall mean ES analysis.

The current study tried to solve the ‘file-drawer problem,' which is frequently connected to meta-analysis. This study calculated Rosenthal (1979) fail-safe N, the Egger test, and the Begg and funnel plots to reduce the impact of the file drawer problem. In Figure 3, the funnel plot is displayed. A visual interpretation of the funnel plot and Egger's test indicates that there is no connection between standard error and the ES. The funnel plot of the eleven-ES, for instance, shows one outlier but no significant asymmetry (Figure 3). In the overall number of studies, the Begg's test for small-study effects and Egger's regression test revealed no indication of publication bias (*p* = 0.352 and *p* = 0.1066, respectively). In addition to that, the fail-safe N of 427 far exceeded the threshold of 115 (z = 11.84, *p* < 0.001; 70 = 5X12+10; 5k+10, Card, 2011). Neither test showed publication bias, so selection modeling was not needed.

### 4.5. Subgroup analyses

The authors conducted meta-regression analysis for continuous variables and subgroup analyses for categorical variables (Table 3) to test for moderators of the interventions' influence on hardiness (Table 4).

**Table 3:** Subgroup comparison results (categorical variables).

**Moderator**	***k***	***g***	**Point estimate SE**	**95% CI**	**Q**	***p***	***I* ^2^**
**Respondent type**	**Q** = **0.508 (*****p*** = **0.476)**						
Med students	4	0.796	0.383	[0.046, 1.545]	67.364^***^	0.000	95.547
Non-Med students	8	1.179	0.339	[0.514, 1.843]	55.304^***^	0.000	87.343
**Culture**	**Q** = 7.519^**^(***p*** = **0.006)**						
Non-Western	9	1.322	0.305	[0.724, 1.920]	72.581^***^	0.000	88.978
Western	3	0.287	0.222	[−0.148, 0.723]	11.044^**^	0.004	81.890
**Intervention types**	**Q** = 8.424^*^(***p*** = **0.015)**						
A cognitive-based intervention	5	2.015	0.503	[1.029, 3.001]	37.235^***^	0.000	89.257
Hardiness interventions	2	0.487	0.470	[0.358, 0.711]	5.939^*^	0.015	83.161
Other interventions	5	0.535	0.090	[−0.435, 1.409]	2.993	0.559	87.000
**Research design**	**Q** = **0.145 (*****p*** = **0.703)**						
Quasi-experimental	6	0.916	0.289	[0.351, 1.482]	36.137^***^	0.000	86.164
Non-quasi-experimental	6	1.039	0.414	[0.228, 1.850]	86.847^***^	0.000	94.243

^*^p < 0.05, ^***^p < 0.001.

**Table 4:** Meta-regression analysis of continuous variables (random-effects model).

**Variable**	**Estimate**	**SE**	***Z***	***p***	**95%CI**	***Q_*M*_***	***df***
Publication year	0.1925^*^	0.172	2.68	0.0075	(0.0515, 0.333)	27.02	1
Duration	0.1423^*^	0.155	2.05	0.0057	(0.111, 0.505)	13.12	1
Q Model (2, k = 12) = 7.23. *p* = 0.0269							

K, number of effect sizes; CI, confidence interval. ^*^p < 0.05.

#### 4.5.1. Respondent type

The respondent type was examined as a moderator. Four studies used medical students and eight studies used non-medical students as respondents. Table 3 shows the results of the subgroup analysis. The ES for the association between hardiness intervention and both types of respondents was significant (*p* < 0.001). However, there were no significant differences detected between respondent types (Q = 0.508, *p* = 0.476).

#### 4.5.2. Culture

The region of origin of the studies also had a significant moderating effect on this outcome (Q = 7.519; *p* = 0.006). Studies from non-Western countries had a better ES (g = 0.305, 95% CI 0.724, 1.920, *p* < 0.001) than studies from Western countries (g = 0.305, 95% CI 0.724, 1.920, *p* < 0.001).

#### 4.5.3. Intervention types

In the included studies, hardiness interventions were done *via* different types of interventions, including cognitive-based intervention (CBI; Toosang et al., 2021), hardiness interventions (Nikoozadeh, 2020), and other types of interventions (Antika et al., 2020). The ES between the cognitive-based intervention and control groups was 0.503 (95% CI 1.029 to 3.001), indicating a medium positive effect. In addition to that, significant subgroup differences between the use of hardiness interventions were found (*I*^2^ = 83%, *p* = 0.015), with a small statistically significant effect = 0.470 (95% CI 0.358–0.711) in favor of the intervention group. However, no significant difference was found in other interventions, (*I*^2^ = 87%, *p* = 0.559), with a small statistically significant effect of 0.090 (95% CI = −0.435, 1.409).

#### 4.5.4. Research design

The included hardiness intervention studies had different research designs, including quasi-experimental and non-quasi-experimental studies. Subgroup analysis indicated that there was a non-significant difference between research designs (Q = 0.145, *p* = 0.703). Conversely, the ESs for both designs were significant.

### 4.6. Meta-regression

To determine if publication year and the length of interventions, which ranged from 4 to 16 weeks, were significant predictors of ES using different models, univariate meta-regression was conducted (Table 4). Results showed that all variables were significant predictors of ES, both publication year [QM (1) = 27.02, *p* < 0.000] and duration [QM (1) = 13.12, *p* = 0.000]. The results imply that the time spent on the interventions and the year of publication are systematically related to the improvement of hardiness.

## 5. Discussion

The concept of hardiness, and whether intervention tactics might improve it among university students, have gained more and more attention. To the best of our knowledge, this is the first meta-analysis that specifically looks at how well various interventions may have changed hardiness, as measured by established hardiness measures. The current findings show that some forms of hardiness intervention seem to be advantageous in the setting of HE. Cognitive-based intervention, in particular, seems to be able to greatly improve measures of hardiness. By examining the impact of intervention techniques in many studies that have a focus on university students, this study contributes to the body of knowledge on hardiness.

The goal of this research was to comprehensively examine the important findings of 12 studies, with 640 participants, that were done till March, 2021, to assess the effectiveness of interventions in improving university students' hardiness. The results of this meta-analysis demonstrated that interventions were efficacious for university students' hardiness. The studies discussed here varied greatly in study design, sample size, and outcome measures. Four studies used one-group (Antika et al., 2020; Khoiriyah et al., 2020; Nikoozadeh, 2020; White et al., 2020). It's interesting to note that only one research found an insignificant improvement in hardiness (White et al., 2020) post-intervention. Half of the studies (6) were quasi-experimental. Five reported statistically significant findings involving increased hardiness (Jafar et al., 2016; Almahaireh et al., 2018; Sahranavard et al., 2019; Torfayeh et al., 2020; Toosang et al., 2021). Only one reported insignificant findings (Jameson, 2014). The present review identified four studies that used CBT and other related techniques (Jafar et al., 2016; Sahranavard et al., 2019; Khoiriyah et al., 2020; Toosang et al., 2021). These studies reported that CBT had a significant influence on improving university students' hardiness.

Overall, a meta-analysis of 12 ESs from 12 empirical researches on enhancing hardiness in college students resulted in a general ES of 0.998, with a *p*-value of 0.000. The 95% CI of the overall ES was (0.549, 1.446). This suggests that intervention strategies affect and increase university students' hardiness and their dimensions (commitment, control, and challenge). According to standard cut-off values, the magnitude of the overall ES is large (Cohen, 1988). The results of this study reflect previous researches' findings that intervention tactics are typically beneficial in promoting students' hardiness (White et al., 2020). This is promising for educators, who are trying to foster this hardiness so students can learn to handle stress appropriately. This result is consistent with the third wave of resilience research, which is concerned with creating and assessing interventions to boost psychological resilience and avoid stress-related mental disorders (Waite and Richardson, 2004; Bengel and Lyssenko, 2012).

After determining the overall ES, the current study checked to see whether there was any significant variation in the study results. A test for heterogeneity was performed for this reason. Results revealed a *p*-value of < 0.001 and a Q-statistic of 123.147. This implies that there was statistically significant variation among ESs from various studies. To put it another way, the ESs of the interventions were inconsistent and dissimilar from one another. This outcome is not unexpected. Of course, the intervention's impact varied from study to study. There always tends to be various true ESs underlying different studies, and, hence, diversity across ESs – unless the ESs concern a series of experiments that were done by the same researchers utilizing the same intervention and similar techniques (Borenstein et al., 2009). Examining potential causes of the variation between ESs was necessary to determine if the test for heterogeneity's significant result was accurate.

Results from the subgroup analyses found that the effects on university students' hardiness were significant for cognitive–behavioral individual or group psychotherapy (CBT) and hardiness interventions, but not for other interventions, like preventive counseling programs and internet-based interventions. Significant subgroup differences between the usage of the cognitive-based therapies (i.e., cognitive restructuring techniques, group training of CBT-based stress management, and metacognitive therapy) were reported in four of the twelve studies. Torfayeh et al. (2020) explored the effects an 8-session course of metacognitive therapy had on improving the psychological hardiness of students. Their findings revealed significant within-group effects on the psychological hardiness measure. In the three phases of pre-test, post-test, and follow-up, for the psychological hardiness variable, there were significant differences between the experimental and control groups (p = 0.001).

Moreover, the intervention had a significant effect on psychological hardiness. Likewise, Toosang et al. (2021) compared the scores of a group of students who received nine interventional sessions of CBT with the scores of subjects in a control group. In the post-test, they discovered that students in the intervention group had much lower levels of depression and anxiety than those in the control group. In the post-training period of CBT, there was a statistically significant difference between the mean scores of hardiness and their dimensions in the two groups. Another study, conducted by Jafar et al. (2016), evaluated an intervention program comprising elements of CBT-based stress management. The randomized controlled design was comprised of two groups that received the Beck Anxiety Inventory. The findings of MANOVA showed that there was a significant difference in the hardiness of the two groups. Using a convenience sample (intervention group) and a control group, Sahranavard et al. (2019) demonstrated group training using cognitive-behavioral therapy-based stress management to improve the hardiness of female students at the Birjand University of Medical Sciences. The study showed that the means of hardiness increased in the post-test for the experimental group. This meta-analysis's results show that cognitive-based interventions can complement or offer an alternative to pharmacological treatments, especially when implemented in the symptomatic stages or when mild cognitive impairment begins to develop. Unwin et al. (2016) found that CBT increases hardiness, which supports this. Students in HE who lack resilience may benefit from CBT interventions.

Furthermore, for hardiness interventions, two of the twelve trials found significant differences between subgroups. Nikoozadeh (2020) investigated whether 12 sessions of hardiness training intervention could lead to significant changes in students' perceived stress and psychological hardiness. The sub-analysis results revealed a substantial decrease in felt stress and an improvement in psychological hardiness in the experimental group, but not so much in the control group. Other studies investigated interventions that included only some aspects of mindfulness. Jameson (2014) tested whether hardiness educational intervention would improve the hardiness and perceived stress levels of junior baccalaureate nursing students. Results revealed that the hardiness intervention had no statistically significant impact on raising the hardiness scores of either group. The study's findings suggest that a hardiness educational intervention may boost undergraduate students' academic performance by teaching them to view stress as an opportunity for growth rather than adversity. According to Maddi et al. (2009), hardiness education is an effective strategy for managing stress. For pupils undergoing stressful transitions, hardiness education supports adaptive coping and provides a road to resilience. Overall, it seems that hardiness training could be successful in providing the knowledge necessary to promote hardiness among students.

Sub-analysis results also revealed that sensitivity to university students' culturally embedded hardiness can be found in both Western and non-Western countries. We highlighted that hardiness is not only a student's capacity to overcome adversity, but also the capacity of the student's environment to provide access to health-enhancing resources in culturally relevant ways. The meta-regression results revealed that a study's publication year was a significant moderator, indicating that earlier studies produced larger ESs than more recent ones; and that the duration of the intervention allows for more opportunities to present information about attitudinal and behavioral change skills, thus allowing participants to reflect on the intervention material between sessions and allowing them to practice new skills. As a result, it is not unexpected that complicated interventions have greater effects. In the current meta-analysis, four trials with intervention durations of 10 weeks or longer indicated a substantial increase in university students' hardiness (Jafar et al., 2016; Sahranavard et al., 2019; Nikoozadeh, 2020; Toosang et al., 2021).

## 6. Conclusion

In a changing world, university students will confront new problems and devise new tactics to keep up with shifting expectations. A significant proportion of young adults and students are seeking psychological services. As a result, the supply of such services is a critical professional concern all over the world. Mental disorders and illnesses are growing increasingly common among students, but college and university counseling facilities are not keeping up with the demand. As such, this is an excellent opportunity to study interventions for university students and determine what is available, what is still needed, and what services may be introduced to guarantee that students' hardiness is addressed. Our findings point to the need to continue to research and promote hardiness among university students, as well as the need to invest in properly educated personnel and, in the long run, establish CBT and hardiness interventions for students who may not otherwise have access to treatment. More comprehensive services, in particular, are required to assist students with mental health issues. Both health and education experts must invest in interventions for university students at strategic locations to develop hardiness. This will address a broader spectrum of unique and complicated student mental health concerns, while also mitigating the unbalanced supply and demand for services.

## 7. Study limitations and future study

Although meta-analysis is becoming more commonly used to assess the effectiveness of interventions, its limits should also be recognized (Butler et al., 2006). The study's small sample size meant that, despite the ES being significantly greater than the others, it had wide CIs and contributed relatively little to the total aggregate impact. Aggregating ESs may hide trial-to-trial variation, but including moderator factors, like in this meta-analysis, may assist in overcoming this drawback. However, due to the small number of studies included, the authors were unable to evaluate every potential moderator concerning participant and facilitator characteristics. For instance, the present meta-analysis did not assess participant gender, the nature of the hardiness interventions, dropout rates, or facilitator adherence or fidelity, all of which might have contributed to the studies' variability and should be included in the future meta-analyses. Notably, both genders were represented among the participants in these investigations. Nonetheless, concentrating on gender-specific methods is an area for further research. Furthermore, because this study only included studies published in peer-reviewed journals in English, we should be wary of the possibility of publication bias, even if our analyses suggest that it is unlikely to be a substantial concern for this meta-analysis. The intervention studies were carried out in several countries; however, the majority of studies were carried out in non-English speaking countries, thus generalizability to other cultural contexts may be restricted. In general, interventions in the included trials were brief, which might reduce the interventions' impacts. As the majority of studies used Iranian samples, generalizability is similarly restricted. Future studies must, therefore, replicate these findings using racially/ethnically varied samples from other nations.

To ascertain if interventions to lessen the quantity or severity of stressors, interventions to lessen the effects of stress, or interventions to reappraise stressors may produce more consistent findings, future research should incorporate a bigger sample size of randomized controlled trials. Furthermore, longitudinal studies are needed to assess if any of the intervention strategies discussed in this study have long-term effectiveness. It is critical to understand which interventions are most operative in decreasing stress in university students in the short term, as well as which interventions have long-term effects on improved comprehension and retention of information, decreased attrition, and retention through an initial exposure to practice in the first year after graduation. Not only will university students and professors benefit, but so will healthcare employers and patients.

## Data availability statement

The raw data supporting the conclusions of this article will be made available by the authors, without undue reservation.

## Author contributions

GJ, ZZa, and SR contributed to the conception and design of the study. SR organized the database. ZZa performed the statistical analysis. ZZa and SR wrote the first draft of the manuscript. NB, SG, ZZa, and SR wrote sections of the manuscript. All authors contributed to manuscript revision, read, and approved the submitted version.

## References

[B1] Abdollahi A. Carlbring P. Vaez E. Ghahfarokhi S. A. (2018a). Perfectionism and test anxiety among high-school students: The moderating role of academic hardiness. Curr. Psychol. 37, 632–639. 10.1007/s12144-016-9550-z

[B2] Abdollahi A. Hosseinian S. Zamanshoar E. Beh-Pajooh A. Carlbring P. (2018b). The moderating effect of hardiness on the relationships between problem-solving skills and perceived stress with suicidal ideation in nursing students. Stud. Psychol. 60, 30–41. 10.21909/sp.2018.01.750

[B3] Abdollahi A. Talib M. A. Yaacob S. N. Ismail Z. (2015). Problem-solving skills appraisal mediates hardiness and suicidal ideation among Malaysian undergraduate students. PLoS ONE 10, e0122222. 10.1371/journal.pone.0122222 25830229 PMC4382337

[B4] Almahaireh A. S. F. Aldalaeen A. S. R. Takhaineh S. K. A. (2018). Efficacy of a preventive counseling program for improving psychological hardiness and the positive use of social network sites among students at risk. Int. J. Adv. Counsell. 40, 173–186. 10.1007/s10447-018-9319-1

[B5] Andrew M. E. Howsare J. L. Charles L. E. McCanlies E. C. Mnatsakanova A. Hartley T. A. . (2013). Associations between protective factors and psychological distress vary by gender: the buffalo cardio-metabolic occupational police stress study. Int. J. Emerg. Ment. Health 15, 277–288. 10.1037/e577572014-032 24707590 PMC4680955

[B6] Antika E. R. Mulawarman M. Mawadah Z. (2020). “Applying mind-skills training to improve academic hardiness on guidance and counseling students with academic burnout,” in 2nd International Seminar on Guidance and Counseling 2019 (ISGC 2019) (Atlantis Press) 89–92. 10.2991/assehr.k.200814.020

[B7] Bakker A. B. de Vries J. D. (2021). Job Demands–Resources theory and self-regulation: New explanations and remedies for job burnout. Anxiety Stress Coping 34, 1–21. 10.1080/10615806.2020.1797695 32856957

[B8] Bartlett D. Cox P. D. (2002). Measuring change in students' critical thinking ability: implications for health care education. J. Allied Health 31, 64–69. 12040999

[B9] Bartone P. T. (1989). Predictors of stress-related illness in city bus drivers. J. Occup. Med. 31, 657–663. 10.1097/00043764-198908000-00008 2668455

[B10] Bartone P. T. (2007). Test-retest reliability of the dispositional resilience scale-15, a brief hardiness scale. Psychol. Rep. 101, 943–944. 10.2466/pr0.101.3.943-944 18232452

[B11] Bartone P. T. (2013). Cross-cultural adaptation of the DRS-15 dispositional resilience scale: a short hardiness measure. Bergen, Norway Available online at at: http://www.hardi-ness-resilience.com/docs/CrossculturaladaptationofDRSFulbright.pdf (accessed July 29, 2020).

[B12] Bartone P. T. Roland R. R. Picano J. J. Williams T. J. (2008). Psychological hardiness predicts success in US army special forces candidates. Int. J. Selec. Assess. 16, 78–81. 10.1111/j.1468-2389.2008.00412.x

[B13] Bartone P. T. Valdes J. J. Sandvik A. (2016). Psychological hardiness predicts cardiovascular health. Psychol. Health Med. 21, 743–749. 10.1080/13548506.2015.1120323 26652199

[B14] Bengel J. Lyssenko L. (2012). Resilienz und psychologische Schutz-faktoren im Erwachsenenalter [Resilience and psychological protective factors in adulthood]. Köln: BzgA.

[B15] Benishek L. A. Lopez F. G. (2001). Development and initial validation of a measure of academic hardiness. J. Career Assess. 9, 333–352. 10.1177/10690727010090040235077271

[B16] Borenstein M. Cooper H. Hedges L. Valentine J. (2009). “Effect sizes for continuous data,” in The handbook of research synthesis and meta-analysis, eds H. Cooper, L. Hedges, and J. C. Valentine (New York, NY: Russell Sage Foundation), 221–235.

[B17] Borenstein M. Hedges L. V. Higgins J. P. Rothstein H. R. (2021). Introduction to Meta-Analysis. 2nd ed. Hoboken, NJ: John Wiley and Sons. 10.1002/9781119558378

[B18] Briggs A. R. Clark J. Hall I. (2012). Building bridges: understanding student transition to university. Qual. Higher Edu. 18, 3–21. 10.1080/13538322.2011.614468

[B19] Britt T. W. Adler A. B. Bartone P. T. (2001). Deriving benefits from stressful events: the role of engagement in meaningful work and hardiness. J. Occup. Health Psychol. 6, 53–63. 10.1037/1076-8998.6.1.53 11199257

[B20] Burbach M. E. Matkin G. S. Fritz S. M. (2004). Teaching critical thinking in an introductory leadership course utilizing active learning strategies: a confirmatory study. Coll. Stud. J. 38, 482–494.

[B21] Butler A. C. Chapman J. E. Forman E. M. Beck A. T. (2006). The empirical status of cognitive-behavioral therapy: a review of meta-analyses. Clin. Psychol. Rev. 26, 17–31. 10.1016/j.cpr.2005.07.003 16199119

[B22] Cheng Y. H. Tsai C. C. Liang J. C. (2019). Academic hardiness and academic self-efficacy in graduate studies. Higher Edu. Res. Develop. 38, 907–921. 10.1080/07294360.2019.1612858

[B23] Cohen J. (1988). Statistical Power Analysis for the Behavioral Sciences. 2nd ed. Hillsdale, NJ: Erlbaum.

[B24] Cole M. S. Feild H. S. Harris S. G. (2004). Student learning motivation and psychological hardiness: Interactive effects on students' reactions to a management class. Acad. Manag. Learn. Educ. 3, 64–85. 10.5465/amle.2004.12436819

[B25] Dale P. M. Ballotti D. (1997). An approach to teaching problem solving in the classroom. Coll. Stud. J. 31, 76–79.

[B26] Fard R. J. Moradkhani F. (2018). Effectiveness of acceptance and commitment therapy on hardiness, procrastination, and frustration tolerance in students of Islamic Azad University, Ahvaz branch, Iran. Int. J. Body Mind Cult. 5, 221–228.

[B27] Hedges L. V. Olkin I. (1985). Statistical Methods for Meta-Analysis. New York, NY: Academic Press.

[B28] Higgins J. P. Thompson S. G. (2002). Quantifying heterogeneity in a meta-analysis. Stat. Med. 21, 1539–1558. 10.1002/sim.1186 12111919

[B29] Huang J. T. (2015). Hardiness, perceived employability, and career decision self-efficacy among Taiwanese college students. J. Career Dev. 42, 311–324. 10.1177/0894845314562960

[B30] Hystad S. W. Eid J. Johnsen B. H. Laberg J. C. Thomas Bartone P. (2010). Psychometric properties of the revised Norwegian dispositional resilience (hardiness) scale. Scand. J. Psychol. 51, 237–245. 10.1111/j.1467-9450.2009.00759.x 20028488

[B31] Jafar H. M. Salabifard S. Mousavi S. M. Sobhani Z. (2016). The effectiveness of group training of CBT-based stress management on anxiety, psychological hardiness and general self-efficacy among university students. Glob. J. Health Sci. 8, 47–54. 10.5539/gjhs.v8n6p47 26755483 PMC4954877

[B32] Jameson P. R. (2014). The effects of a hardiness educational intervention on hardiness and perceived stress of junior baccalaureate nursing students. Nurse Educ. Today 34, 603–607. 10.1016/j.nedt.2013.06.019 23870691

[B33] Johnsen B. H. Bartone P. Sandvik A. M. Gjeldnes R. Morken A. M. Hystad S. W. . (2013). Psychological hardiness predicts success in a n orwegian armed forces border patrol selection course. Int. J. Select. Assess. 21, 368–375. 10.1111/ijsa.12046

[B34] Johnsen B. H. Espevik R. Saus E.-R. Sanden S. Olsen O. K. Hystad S. W. (2017). Hardiness as a moderator and motivation for operational duties as mediator: The relation between operational self-efficacy, performance satisfaction, and perceived strain in a simulated police training scenario. J. Pol. Crim. Psychol. 32, 331–339. 10.1007/s11896-017-9225-1

[B35] Johnson W. L. Giordano P. C. Manning W. D. Longmore M. A. (2011). Parent–child relations and offending during young adulthood. J. Youth Adolesc. 40, 786–799. 10.1007/s10964-010-9591-9 20865307 PMC3112466

[B36] Kanekar A. Sharma M. Atri A. (2010). Enhancing social support, hardiness, and acculturation to improve mental health among Asian Indian international students. Int. Q. Community Health Educ. 30, 55–68. 10.2190/IQ.30.1.e 20353927

[B37] Khoiriyah Z. Sugiharto D. Y. P. Japar M. (2020). The effectiveness of group counseling with self-instruction and cognitive restructuring techniques to improve hardiness. Jurnal Bimbingan Konseling 9, 152–157.

[B38] Kmet L. M. Cook L. S. Lee R. C. (2004). Standard quality assessment criteria for evaluating primary research papers from a variety of fields. Alberta Heritage Foundation for Medical Research Available online at: https://era.library.ualberta.ca/items/48b9b989-c221-4df6-9e35-af782082280e (accessed August 16, 2019).

[B39] Kobasa S. C. (1979). Stressful life events, personality, and health: an inquiry into hardiness. J. Pers. Soc. Psychol. 37, 1–11. 10.1037/0022-3514.37.1.1 458548

[B40] Kobasa S. C. Maddi S. R. Kahn S. (1982). Hardiness and health: a prospective study. J. Pers. Soc. Psychol. 42, 168–177. 10.1037//0022-3514.42.1.168 7057354

[B41] Kowalski C. M. Schermer J. A. (2019). Hardiness, perseverative cognition, anxiety, and health-related outcomes: a case for and against psychological hardiness. Psychol. Rep. 122, 2096–2118. 10.1177/0033294118800444 30253687

[B42] Lang A. Goulet C. Amsel R. (2003). Lang and Goulet hardiness scale: Development and testing on bereaved parents following the death of their fetus/infant. Death Stud. 27, 851–880. 10.1080/716100345 14610777

[B43] Lazarus R. S. (2000). “Evolution of a model of stress, coping, and discrete emotions,” in Handbook of Stress, Coping, and Health: Implications for Nursing Research, Theory, and Practice, ed. V. H. Rice (Los Angeles, CA: Sage) 195–222.

[B44] Light R. J. Pillemer D. B. (1984). Summing up: The Science of Reviewing Research. Cambridge, MA: Harvard University Press. 10.4159/9780674040243

[B45] Lipsey M. W. Wilson D. B. (2001). Practical Meta-Analysis. Thousand Oaks, CA: SAGE publications, Inc.

[B46] Macedo T. Wilheim L. Gonçalves R. Coutinho E. S. F. Vilete L. Figueira I. . (2014). Building resilience for future adversity: a systematic review of interventions in non-clinical samples of adults. BMC Psychiatry 14, 1–8. 10.1186/s12888-014-0227-6 25266031 PMC4149241

[B47] Maddi S. R. (1987). “Hardiness training at Illinois Bell Telephone,” in Health Promotion Evaluation, ed. J. Opatz (Stephens Point, WI: National Wellness Institute) 101–115.

[B48] Maddi S. R. (1999). The personality construct of hardiness: I. Effects on experiencing, coping, and strain. Consult. Psychol J. Pract. Res. 51, 83–94. 10.1037/1061-4087.51.2.83

[B49] Maddi S. R. (2002). The story of hardiness: twenty years of theorizing, research, and practice. Consult. Psychol. J. Pract. Res. 54, 173. 10.1037/1061-4087.54.3.173

[B50] Maddi S. R. Harvey R. H. (2006). “Hardiness considered across cultures,” in Handbook of Multicultural Perspectives on Stress and Coping (Boston, MA: Springer), 409–426. 10.1007/0-387-26238-5_17

[B51] Maddi S. R. Harvey R. H. Khoshaba D. M. Fazel M. Resurreccion N. (2009). Hardiness training facilitates performance in college. J. Posit. Psychol. 4, 566–577. 10.1080/17439760903157133

[B52] Maddi S. R. Kahn S. Maddi K. L. (1998). The effectiveness of hardiness training. Consult. Psychol. J. Pract. Res. 50, 78–86. 10.1037/1061-4087.50.2.78

[B53] Maddi S. R. Khoshaba D. M. (2001). Personal Views Survey III-R: Test Development and Internet Manual. Newport Beach, CA: Hardiness Institute.

[B54] Maddi S. R. Khoshaba D. M. Jensen K. Carter E. Lu J. L. Harvey R. H. (2002). Hardiness training for high-risk undergraduates. NACADA J. 22, 45–55. 10.12930/0271-9517-22.1.45

[B55] Maddi S. R. Matthews M. D. Kelly D. R. Villarreal B. White M. (2012). The role of hardiness and grit in predicting performance and retention of USMA cadets. Military Psychol. 24, 19–28. 10.1080/08995605.2012.639672

[B56] Magnussen L. Ishida D. Itano J. (2000). The impact of the use of inquiry-based learning as a teaching methodology on the development of critical thinking. J. Nurs. Edu. 39, 360–364. 10.3928/0148-4834-20001101-07 11103974

[B57] Martens R. (1987). Coaches Guide to Sport Psychology. Champaign, IL: Human Kinetics.

[B58] McCubbin M. A. McCubbin H. I. Thompson A. I. (1986). “Family Hardiness Index (FHI),” in Family Assessment: Resiliency, Coping and Adaptation Inventories for Research and Practice, eds. H. I. McCubbin, A. I. Thompson, and M. A. McCubbin (Madison, WI: University of Wisconsin±Madison) 239–305.

[B59] McGregor G. D. (2001). Creative Thinking Instruction for a College Study Skills Program: A Case Study. Waco, TX.

[B60] McKown C. (2004). Age and ethnic variation in children's thinking about the nature of racism. J. Appl. Dev. Psychol. 25, 597–617. 10.1016/j.appdev.2004.08.001

[B61] Moher D. Liberati A. Tetzlaff J. Altman D. G. Group,^*^ P. (2009). Preferred reporting items for systematic reviews and meta-analyses: the PRISMA statement. Ann. Intern. Med. 151, 264–269. 10.7326/0003-4819-151-4-200908180-00135 19622511

[B62] Nikoozadeh E. K. (2020). Effectiveness of Hardiness Training Intervention on Students' Perceived Stress and Psychological Hardiness. Int. J. Appl. Behav. Sci. 7, 58–66.

[B63] Pollock S. E. (1986). Human responses to chronic illness: physiologic and psychosocial adaptation. Nurs. Res. 35, 90–95. 10.1097/00006199-198603000-00008 3633512

[B64] Ribeiro I. J. Pereira R. Freire I. V. de Oliveira B. G. Casotti C. A. Boery E. N. (2018). Stress and quality of life among university students: a systematic literature review. Health Profess. Edu. 4, 70–77. 10.1016/j.hpe.2017.03.002

[B65] Rosenthal R. (1979). The file drawer problem and tolerance for null results. Psychol. Bull. 86, 638–641. 10.1037/0033-2909.86.3.638

[B66] Sahranavard S. Esmaeili A. Salehiniya H. Behdani S. (2019). The effectiveness of group training of cognitive behavioral therapy-based stress management on anxiety, hardiness and self-efficacy in female medical students. J. Educ. Health Promot. 8, 1–7. 10.1051/bmdcn/2018080423 30993142 PMC6432834

[B67] Scott J. N. Markert R. J. Dunn M. M. (1998). Critical thinking: change during medical school and relationship to performance in clinical clerkships. Med. Educ. 32, 14–18. 10.1046/j.1365-2923.1998.00701.x 9624394

[B68] Spelic S. S. Parsons M. Hercinger M. Andrews A. Parks J. Norris J. (2001). Evaluation of critical thinking outcomes of a BSN program. Holist. Nurs. Pract. 15, 27–34. 10.1097/00004650-200104000-00007 12120108

[B69] Stein S. J. Bartone P. T. (2020). Hardiness: Making Stress Work for You to Achieve Your Life Goals. Hoboken, NJ: John Wiley and Sons.

[B70] Stephens T. M. Gunther M. E. (2016). Twitter, millennials, and nursing education research. Nurs. Educ. Perspect. 37, 23–27. 10.5480/14-1462 27164773

[B71] Taylor Z. E. Doane L. D. Eisenberg N. (2014). Transitioning from high school to college: Relations of social support, ego-resiliency, and maladjustment during emerging adulthood. Emerging Adulthood 2, 105–115. 10.1177/2167696813506885

[B72] Thalheimer W. Cook S. (2002). How to calculate effect sizes from published research: a simplified methodology. Work Learn. Res. 1, 1–9.

[B73] Thomas N. (2012). Love, rights and solidarity: Studying children's participation using Honneth's theory of recognition. Childhood 19, 453–466. 10.1177/0907568211434604

[B74] Thompson C. Rebeschi L. M. (1999). Critical thinking skills of baccalaureate nursing students at program entry and exit. Nurs. Health Care Perspect. 20, 248–248. 10754847

[B75] Toosang M. A. Pasha R. Safarzadeh S. (2021). The effect of cognitive-behavioral therapy training on resilience and psychological hardiness in students during COVID-19 pandemic situation. Int. J. School Health 8, 247–256.

[B76] Torfayeh M. Esmaeili M. Pourabadei P. Yazdani M. (2020). Effectiveness of metacognitive therapy on psychological hardiness of students. Health Edu. Health Promot. 8, 101–105.

[B77] Travis J. Kaszycki A. Geden M. Bunde J. (2020). Some stress is good stress: the challenge-hindrance framework, academic self-efficacy, and academic outcomes. J. Educ. Psychol. 112, 1632–1643. 10.1037/edu0000478

[B78] Unwin G. Tsimopoulou I. Kroese B. S. Azmi S. (2016). Effectiveness of cognitive behavioural therapy (CBT) programmes for anxiety or depression in adults with intellectual disabilities: A review of the literature. Res. Dev. Disabil. 51, 60–75. 10.1016/j.ridd.2015.12.010 26803286

[B79] Viechtbauer W. (2007). Accounting for heterogeneity *via* random-effects models and moderator analyses in meta-analysis. Zeitschrift für Psycho. J. Psychol. 215, 104–121. 10.1027/0044-3409.215.2.104

[B80] Waite P. J. Richardson G. E. (2004). Determining the efficacy of resiliency training in the work site. J. Allied Health 33, 178–183. 15503750

[B81] Wardani R. (2020). Academic hardiness, skills, and psychological well-being on new student. J. Psikol. 19, 188–200. 10.14710/jp.19.2.188-200

[B82] Weissinger P. A. (2003). Critical Thinking Skills of First-Year Dental Students Enrolled in a Hybrid Curriculum With a Problem-Based Learning Component. Bloomington, IN.

[B83] White A. Zapata I. Lenz A. Ryznar R. Nevins N. Hoang T. N. . (2020). Medical students immersed in a hyper-realistic surgical training environment leads to improved measures of emotional resiliency by both hardiness and emotional intelligence evaluation. Front. Psychol. 11, 1–8. 10.3389/fpsyg.2020.569035 33329208 PMC7714941

[B84] Wrench A. Garrett R. King S. (2014). Managing health and well-being: student experiences in transitioning to higher education. Asia-Pacific J. Health Sport Phys. Edu. 5, 151–166. 10.1080/18377122.2014.906059

[B85] Yang B. Guan Q. Huang J. Wang Z. (2022). Peer victimization and nonsuicidal self-injury among Chinese left-behind children: mediation by perceived discrimination and moderation by hardiness. J. Aggre. Maltreat. Trauma 1–17. 10.1080/10926771.2022.2101408

